# Prevention of Wear Particle-Induced Osteolysis by a Novel V-ATPase Inhibitor Saliphenylhalamide through Inhibition of Osteoclast Bone Resorption

**DOI:** 10.1371/journal.pone.0034132

**Published:** 2012-04-11

**Authors:** An Qin, Tak S. Cheng, Zhen Lin, Lei Cao, Shek M. Chim, Nathan J. Pavlos, Jiake Xu, Ming Hao Zheng, Ke Rong Dai

**Affiliations:** 1 Shanghai Key Laboratory of Orthopaedic Implant, Department of Orthopaedics, Ninth People's Hospital, Shanghai Jiao Tong University School of Medicine, Shanghai, The People's Republic of China; 2 Centre for Orthopaedic Research, School of Surgery, The University of Western Australia, Western Australia, Australia; 3 Division of Orthopaedic, Department of Surgery, Guangdong Academy of Medical Sciences, Guangdong General Hospital, Guangdong, The People's Republic of China; 4 School of Pathology and Laboratory Medicine, The University of Western Australia, Western Australia, Australia; 5 Orthopaedic Cellular and Molecular Biology Laboratory, Institute of Health Sciences, School of Medicine, Chinese Academy of Sciences, Shanghai Jiao Tong University, Shanghai, The People's Republic of China; Institut de Génomique Fonctionnelle de Lyon, France

## Abstract

Wear particle-induced peri-implant loosening (Aseptic prosthetic loosening) is one of the most common causes of total joint arthroplasty. It is well established that extensive bone destruction (osteolysis) by osteoclasts is responsible for wear particle-induced peri-implant loosening. Thus, inhibition of osteoclastic bone resorption should prevent wear particle induced osteolysis and may serve as a potential therapeutic avenue for prosthetic loosening. Here, we demonstrate for the first time that saliphenylhalamide, a new V-ATPase inhibitor attenuates wear particle-induced osteolysis in a mouse calvarial model. *In vitro* biochemical and morphological assays revealed that the inhibition of osteolysis is partially attributed to a disruption in osteoclast acidification and polarization, both a prerequisite for osteoclast bone resorption. Interestingly, the V-ATPase inhibitor also impaired osteoclast differentiation via the inhibition of RANKL-induced NF-κB and ERK signaling pathways. In conclusion, we showed that saliphenylhalamide affected multiple physiological processes including osteoclast differentiation, acidification and polarization, leading to inhibition of osteoclast bone resorption *in vitro* and wear particle-induced osteolysis *in vivo*. The results of the study provide proof that the new generation V-ATPase inhibitors, such as saliphenylhalamide, are potential anti-resorptive agents for treatment of peri-implant osteolysis.

## Introduction

Total joint arthroplasty (TJA) is the gold standard for the treatment of end-stage joint diseases such as osteoarthritis and rheumatoid arthritis [1]. Globally, approximately 1.5 million cases of total arthroplasty are carried out per annum [2]. Although extensive efforts have been made to improve the efficacy and quality of TJA, there are still numerous caveats that limit the long-term success of arthroplasty surgery. Aseptic prosthetic loosening is one of the most common causes of TJA. It occurs secondary to a cascade of chronic inflammatory events stimulated and maintained by wear particles [3], [4], [5], [6]. The underlying mechanisms of particle-induced osteolysis are complex, involving numerous cytokines, chemokines, growth factors, and cell types. Wear particles stimulate macrophages, fibroblasts, foreign body giant cells, and T lymphocytes to release vast arrays of proinflammatory cytokines and chemokines including tumour necrosis factor-a (TNFα), interleukins-1, 6, 11 and 17 (IL-1, -6, -11, -17), prostaglandin E_2_ (PGE_2_) and macrophage-colony stimulating factor (M-CSF) all of which induce receptor activator of nuclear factor-κ B ligand (RANKL) expression by osteoblasts, marrow stromal cells and activated T-cells [4], [7], [8], [9], [10], [11], [12], [13], [14], [15], [16]. Increased RANKL levels at the implant site exacerbates the differentiation and activation of the already abundant pool of monocyte/macrophage precursors surrounding the prosthetic implant into mature osteoclasts thus shifting the local homeostasis to activated bone destruction [17], [18], [19], [20], [21].

Given that the central mechanism of wear particle-induced osteolysis is increased osteoclastic bone resorption, therapeutic targeting of osteoclast function presents a logical avenue towards treating or alleviating aseptic loosening after total joint replacement. V-ATPases are distinguished enzyme complexes that differentiate osteoclasts from macrophages and other cells. These proton pumps play vital roles in osteoclastic bone resorption by mediating extracellular acidification of the resorption pit between the osteoclast ruffled border plasma membrane and the bone surface [22], [23], [24]. The critical importance of V-ATPases is exemplified by mutational or knockout genetic studies in humans and mice of specific V-ATPase subunits (e.g. *a3*) exhibiting osteopetrotic phenotypes owing to impaired osteoclastic function [25], [26], [27], [28], [29], [30], [31], [32]. To this end, we speculated that agents targeting V-ATPase could represent a potentially new class of anti-resorptive therapeutics for the treatment of particle-induced osteolysis after total joint replacement.

Although several V-ATPase inhibitors, such as the plecomacrolide antibiotics bafilomycin and concanamycin, have been shown to be effective in inhibiting osteoclast function [33], the ionophoric properties and the lack of specificity on V-ATPase cause systemic unacceptable toxicity when administered to animals. Currently these V-ATPase inhibitors have been precluded from the pharmacological use *in vivo* [34]. Salicylihalamide A (saliA), a marine metabolite represents a new generation of novel V-ATPase inhibitors that exhibit its specific inhibition profile and unique selectivity towards mammalian V-ATPases [34]. SaliA acts on the V0 domain of the V-ATPase complex via a mode of action that is distinct from the other two macrolides, bafilomycin and concanamycin [35]. Here, we have shown for the first time that saliphenylhalamide (1) effectively attenuates titanium particle-induced osteolysis in mouse calvaria *in vivo*; (2) potently inhibits osteoclast acidification function and polarization *in vitro*; and (3) impairs osteoclast formation via disruption of NF-κB and ERK signalings, an unexpected feature that has yet to be assigned to V-ATPase inhibitors.

## Results

### SaliPhe is a potent inhibitor of the osteoclastic V-ATPase acidification machinery

Bone resorption by osteoclasts is strictly dependent on the V-ATPase-mediated acidification machinery. Thus, selective inhibitors of the V-ATPase complex hold great promise for targeted anti-resorptive therapy. As an initial step towards investigating the therapeutic potential of saliPhe as a new anti-resorptive agent we first compared its ability of blocking intracellular acidification in osteoclasts with bafilomycin, an established inhibitor of the osteoclastic V-ATPase complex. For this purpose, acridine orange (AO) fluorescence quenching assays were performed on mature osteoclasts derived from mouse bone marrow macrophages (BMM) stimulated with M-CSF and RANKL (5-days) and then exposed to either the presence or absence of saliPhe or bafilomycin at varying concentrations. AO quenching has been routinely employed to monitor the intracellular acidification status of a variety of cells and organelles [25]. Upon entering acidified compartments, unprotonated AO (green light; Em535 nm) becomes protonated and emits orange/red light (Em580 nm) when excited by blue light (Ex492 nm). Fluorescence shift from green to orange/red indicates normal intracellular acidification.

**Figure 1:**
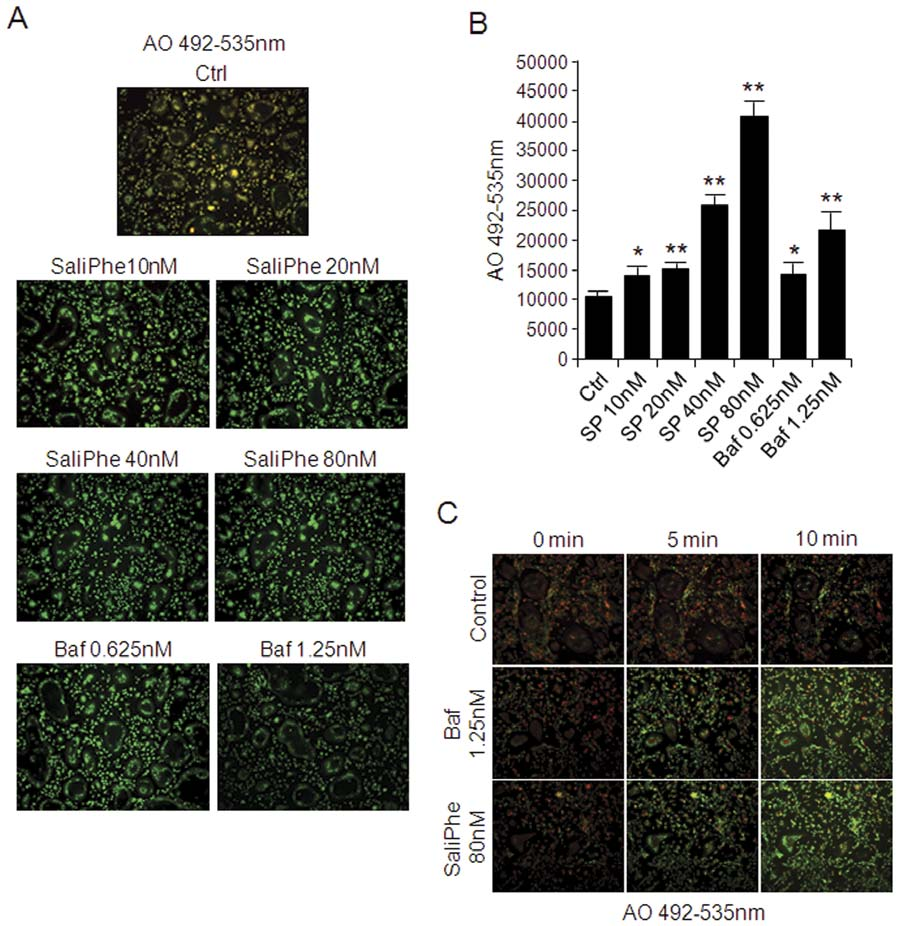
SaliPhe and bafilomycin potently attenuate intracellular acidification in osteoclasts *in vitro*. (**A**) Fluorescence quenching of acridine orange (AO) in BMM-derived osteoclasts following saliPhe or bafilomycin treatment. Mouse BMM-derived osteoclasts were pre-treated for 24 hrs with various concentrations of saliPhe or bafilomycin prior to incubation with 5 µg/ml AO for 30 mins at 37°C. Fluorescence quenching was measured with excitation 492 nm and emission 535 nm. Fluorescence shift of acridine orange from green to orange/red indicate normal intracellular acidification. Overlay (orange/yellow) of red and green fluorescence spectra are presented. (**B**) Acridine orange readings of the green wavelength (Ex492 nm and Em535 nm) was measured in live osteoclasts using the FLUOStar Optima spectrophotometer (BMG LabTech) showing the effect of saliPhe and bafilomycin. The results are representative of three independent experiments performed in triplicates (mean ± SEM). (C) Acridine orange quenching assay in osteoclasts. The mature osteoclasts were incubated with 5 µg/ml AO for 30 mins followed by adding V-ATPase inhibitors for observing acridine orange quenching at 0, 5 and 10 min under fluorescent microscope. The asterisks indicate significant differences between the inhibitors and control (*P<0.05, **P<0.01).

As shown in Figure 1A, cells treated with saliPhe (10 nM, 20 nM, 40 nM, and 80 nM) or bafilomycin (0.625 nM, 1.25 nM) dose-dependently inhibited AO quenching as evidenced by the predominantly green fluorescence signal of the Ex492/Em535 fluorescence spectra, and little shift towards the orange/red fluorescence observed, reflecting reduced acidity in the treated osteoclasts. In contrast, osteoclasts treated with vehicle alone displayed typical intracellular acidification spectra as demonstrated by the fluorescence shift of AO fluorescence from green to orange/red (orange/yellow spectra when merged) (Fig. 1A, control). Additionally, the inhibitory effect on acidification can be observed as early as 5 and 10 mins following treatment of cells with either saliPhe or bafilomycin (Fig. 1C). Quantification of AO fluorescence intensity at Ex492/Em535 using a spectrophotometer further confirmed the dose-dependent elevation of intracellular pH levels (decrease acidity) by saliPhe and bafilomycin (Fig. 1B; *P<0.05, **P<0.01). Taken together, these data indicate a clear impairment in intracellular acidification in osteoclasts following saliPhe treatment, suggesting that saliPhe may be a potent inhibitor of the osteoclastic acidification machinery.

### V-ATPase inhibitors prevented wear particles-induced osteolysis in vivo

**Figure 2:**
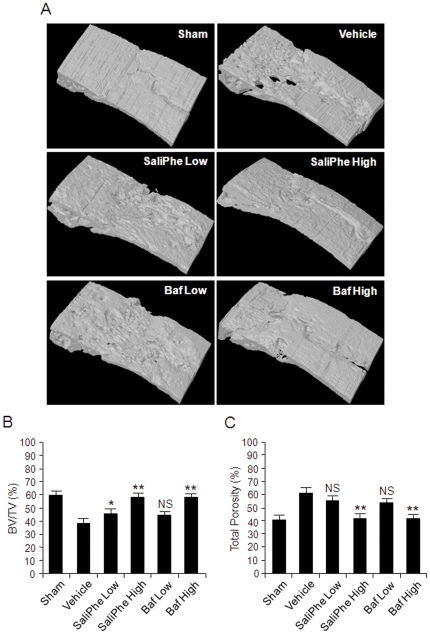
Prevention of wear particle-induced osteolysis *in vivo* by saliPhe and bafilomycin – μCT analysis. (**A**) Representative μCT 3D reconstruction images of selected focal area on the middle suture of mice calvaria from sham, wear particle-induced osteolysis group (vehicle), saliPhe treated group (low dose - 250 nM; or high dose - 500 nM), and bafilomycin treated group (low dose - 100 nM; or high dose - 250 nM). Osseous property analysis from each group was measured from the selected focal area of the middle suture. (**B and C**) The amount of bone mass (% BV/TV) and the amount of bone resorption volume expressed as a percentage of porosity of the whole calvaria (% Total Porosity) was measured. The asterisks indicate significant differences between the inhibitors and vehicle control (*P<0.05, **P<0.01).

Having established that saliPhe is indeed a specific inhibitor of V-ATPase-mediated acidification in osteoclasts we next explored its potential protective effects under pathological settings of osteolysis. To this end, we utilized a titanium particle-induced mouse calvarial osteolysis model to directly compare the effects of saliPhe and bafilomycin on localized particle-induced osteolysis. To achieve this, thirty micrograms of titanium wear particle was embedded under the periosteum at the middle suture of the calvaria in 8-week-old C57BL/J6 mice treated without or with saliPhe (250 nM and 500 nM) or bafilomycin (100 nM and 250 nM). Importantly, no fatalities occurred during or after particle implantation and mice retained normal activity throughout the duration of the experiment. *In vivo* toxicity of bafilomycin and saliPhe has been previously reported [36]. After 14-days, the mice were sacrificed and the degree of particle-induced osteolysis was assessed using high-resolution μCT and histology. As expected, implantation of titanium wear particles induced severe osteolysis as evidenced by the extensive eroded surface observed on the calvaria (vehicle; PBS injection) when compared to negative control (sham; no titanium particles) (Figure 2A). In contrast, treatment of either saliPhe and/or bafilomycin led to a significant reduction in the extent of wear particle-induced bone destruction, particularly at higher doses (500 nM of saliPhe and 250 nM of bafilomycin) (Fig. 2A). Quantitative analysis of bone parameters further confirmed the wear particle–induced osteolysis with a significantly reduction in BV/TV (Fig. 2B; *P<0.05, **P<0.01) and significant increase in total bone porosity of the calvaria (Fig. 2C; **p<0.01).

**Figure 3:**
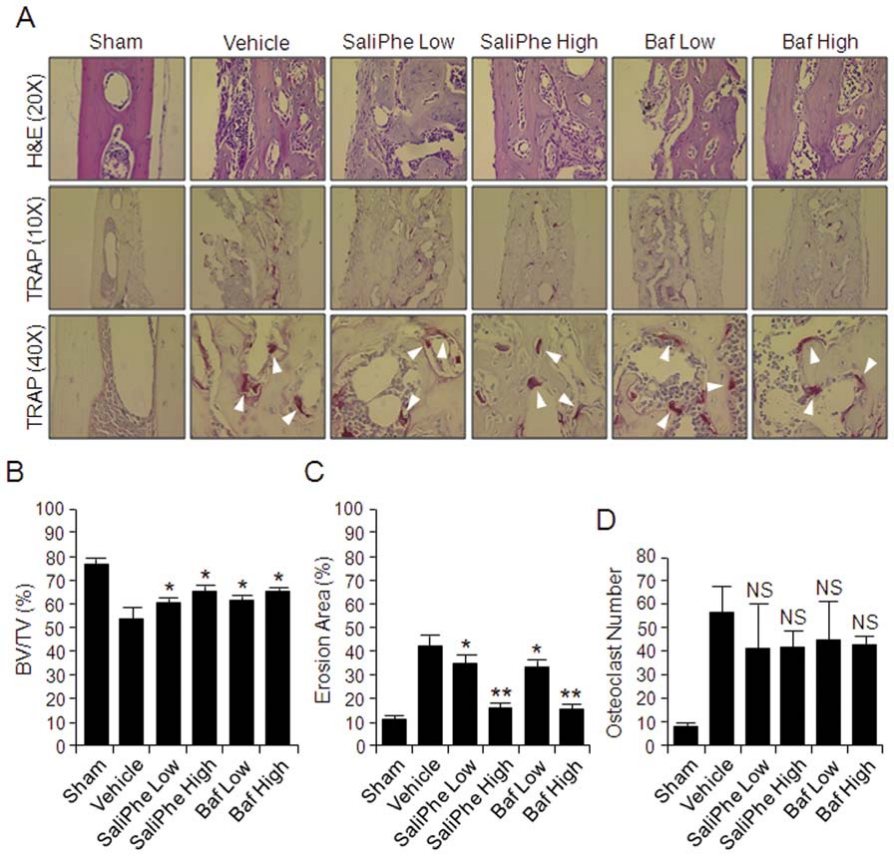
SaliPhe and bafilomycin protect against wear particle-induced osteolysis *in vivo*– histological and histomorphometric analysis. (**A**) Histological H&E appearance of murine calvarial tissues along the middle suture. Both H&E and TRAP staining were taken for at least 3 sections for each group. The sham section shows few inflammatory and osteolytic changes. The vehicle group shows marked inflammatory reaction and prominent osteolysis, whereas the inhibitor treated groups reveal a reduction in both inflammation and osteolysis. White arrowheads indicate TRAP positive osteoclasts. (**B–D**) Histomorphometric analysis of bone volume against tissue volume (% BV/TV), erosion area (the percentage of infiltration fibrotic area against total tissue area), and the number of TRAP positive multinucleated osteoclasts (>3 nuclei) on the bone surface was measured (*P<0.05, **P<0.01 against vehicle control).

Histological H&E assessment and histomorphometric analysis further confirmed the attenuation of wear particle-induced bone erosion by both saliPhe and bafilomycin (Fig. 3A). In this instance, wear particle injection induced an inflammatory infiltration of lymphocyte and macrophages into the site of injection, as well as multiple osteoclasts lining the eroded bone surface as revealed by staining for the osteoclast marker enzyme tartrate-acid resistant phosphatase (TRAP) (Fig. 3A; white arrowheads). Consistent with the μCT quantitation, histomorphometric analysis demonstrated that both low and high dose of saliPhe and bafilomycin significantly reduced the extent of bone erosion induced by the titanium particles (*P<0.05, **P<0.01) additionally with a trend of decrease in osteoclast numbers (Fig. 3B, C, D). Collectively, these data imply that osteoclast resorption function, rather than osteoclast formation rates, were primarily disrupted by both V-ATPase inhibitors *in vivo* (Fig. 3A and D), attesting to the notion that V-ATPase inhibitors like saliPhe serves as effective anti-resorptive agents for the treatment and/or inhibition of particle-induced osteolysis.

### SaliPhe and bafilomycin impaired osteoclast bone resorption in vitro

**Figure 4:**
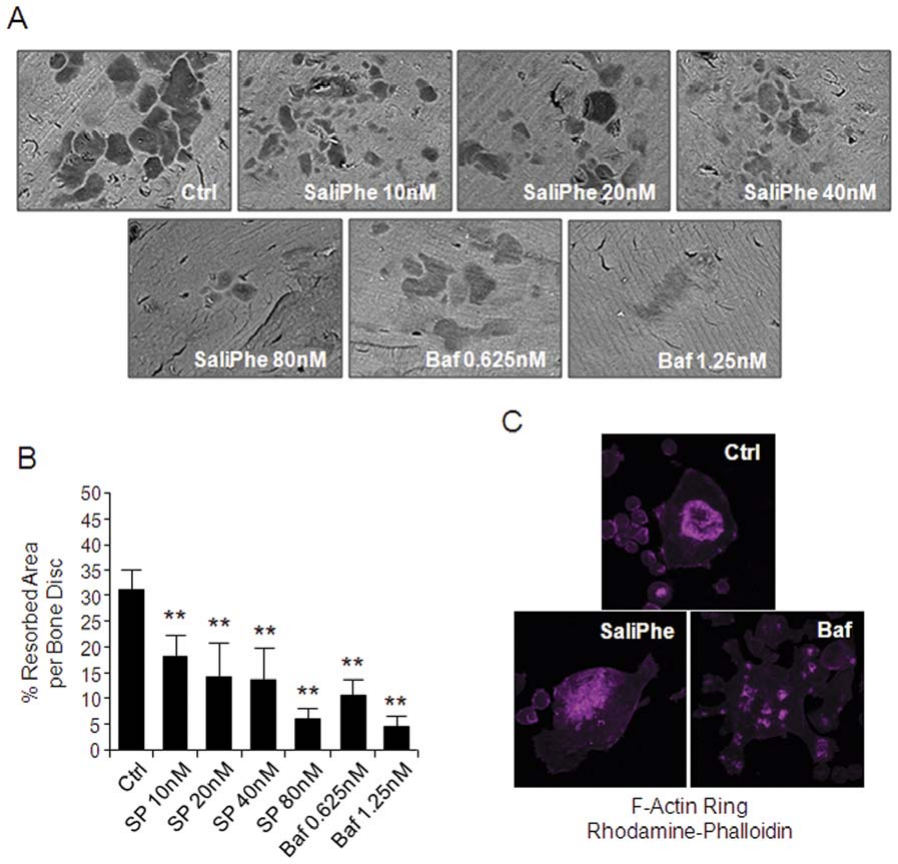
SaliPhe and bafilomycin inhibit osteoclastic bone resorption *in vitro*. (**A**) Pre-osteoclasts cultured on bone discs were incubated in the presence or absence of saliPhe (10 nM, 20 nM, 40 nM, or 80 nM) or bafilomycin (0.625 nM or 1.25 nM) for 48 hrs for assessment of effect on bone resorption. Bone resorption pits were examined by scanning electron microscopy (SEM). (**B**) The resorbed area on each bone disc was quantified as a percentage of the total bone disc area. Data representative of 3 independent experiments performed in triplicates (mean ± SEM; *P<0.05, **P<0.01 against control). (**C**) F-actin ring formation in activated osteoclasts. Osteoclasts cultured on bone discs and treated with saliPhe (80 nM) or bafilomycin (1.25 nM) were stained for the formation of F-actin ring with Alexa-Fluor 647 phalloidin and imaged on NIKON confocal system with 40× oil emersion lens.

Next, we assessed the direct effect of saliPhe and bafilomycin on osteoclast bone-resorptive function *in vitro* using osteoclasts derived from mouse BMMs. BMM-derived pre-osteoclasts stimulated with M-CSF and RANKL for 3 days were cultured on devitalized bovine bone discs in either the presence or absence of various concentrations of the respective V-ATPase inhibitors and then examined for resorption pit formation capacity 48-hrs post-culture. As revealed by scanning electron microscopy (SEM), at doses from 10 nM, saliPhe effectively inhibited osteoclast-mediated bone resorption (∼50%) with almost completely blockade of bone resorption attained at higher concentrations (80 nM) (Fig. 4A and B; **P<0.01). Comparatively, bafilomycin exhibited higher potency for bone resorption inhibition i.e. ∼65% inhibition at 0.625 nM and almost complete abolishment of bone resorption at 1.25 nM (Fig. 4A and B; **P<0.01).

Since the formation of a well-polarized F-actin ring/sealing zone is an essential prerequisite for efficient bone resorption by osteoclasts we also explored the effect of saliPhe and bafilomycin of F-actin ring formation. As expected, characteristic podosomal condensation and F-actin ring formation was observed in untreated control (Ctrl) osteoclasts as visualized by Phalloidin-Alexa Fluor 647 staining and confocal microscopy (Figure 4C). On the other hand, treatment with either saliPhe or bafilomycin resulted in drastic alterations in F-actin ring formation and morphology. In this instance, F-actin tended to aggregate as small pleiomorphic rings that appeared largely unstructured and often varied in both size and number after either saliPhe or bafilomycin treatment (Fig. 4C). Given the fact that SaliPhe and Bafilomycin bind to the V0 domain [37], [38] while direct interaction between V-ATPase and F-actin ring only occurs in B2 and C1 of V1 domain [25], [39], thus we speculated that these two inhibitors may indirectly disrupt F-actin ring formation via the V1 domain. In support of this, bioluminescence resonance energy transfer (BRET) assay showed that the disruption of F-actin ring is at least not due to the alterations in V0 subunits by saliPhe or bafilomycin treatment (Figure S1A). It is noteworthy that B2 and C1 expression level is also not altered by saliPhe or Bafilomycin treatment (Figure S1B and S1C). Taken together, while SaliPhe and bafilomycin exert clear effects on F-actin ring formation, these changes appear to be independent, at least in part, from the V-ATPase V1/V0 expression and assembly. Further studies will be required to define the exact mechanism underlying these cytosketelal aberrations.

### SaliPhe and bafilomycin inhibits osteoclast differentiation in vitro

**Figure 5:**
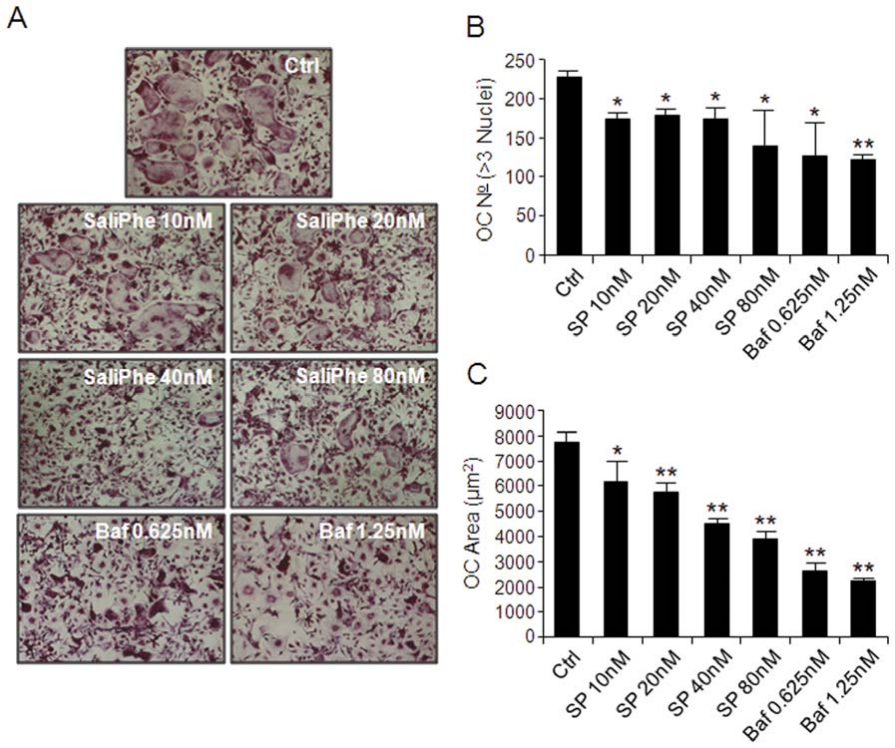
SaliPhe and bafilomycin impair osteoclast formation *in vitro*. (**A**) M-CSF-dependent BMMs were stimulated with 100 ng/ml RANKL in the presence or absence of saliPhe (10 nM, 20 nM, 40 nM, or 80 nM) or bafilomycin (0.625 nM or 1.25 nM) for 5 days for the formation of multinucleated osteoclasts. Cells were fixed and stained for TRAP activity. The number (**B**) and average size in areas (µm^2^) (**C**) of TRAP positive multinucleated osteoclasts (>3 nuclei) were quantified. Data representative of 3 independent experiments performed in triplicates (mean ± SEM; *P<0.05, **P<0.01 against control).

V-ATPase-driven acidification and specific V-ATPase subunits have been recently implicated in the modulation osteoclastogenesis [29], [40]. Therefore, we further explored whether blockade of V-ATPase-mediated acidification by saliPhe and bafilomycin might also affect RANKL-induced osteoclast formation. To test this, murine M-CSF-dependent BMMs were treated with 100 ng/ml RANKL for 5 days to form osteoclasts in the absence or presence of varying concentrations of saliPhe (10 nM, 20 nM, 40 nM and 80 nM) or bafilomycin (0.625 nM and 1.25 nM). As shown in figure 5, both saliPhe and bafilomycin significantly reduced the number of TRAP-positive multinucleated osteoclast (>3 nuclei), up to 25% and 40%, respectively at maximal dosages i.e. 80 nM saliPhe and 1.25 nM bafilomycin (Fig. 5A and B; *P<0.05, **P<0.01). Interestingly, the size of the osteoclasts formed in the presence of both V-ATPase inhibitors were significantly smaller (*P<0.05, **P<0.01) as compared to the RANKL-treated control (Fig. 5C), consistent with previous reports implicating V-ATPases in the regulation of osteoclast formation [29], [40].

### SaliPhe and bafilomycin impair RANKL-induced NF-κB signaling pathway

**Figure 6:**
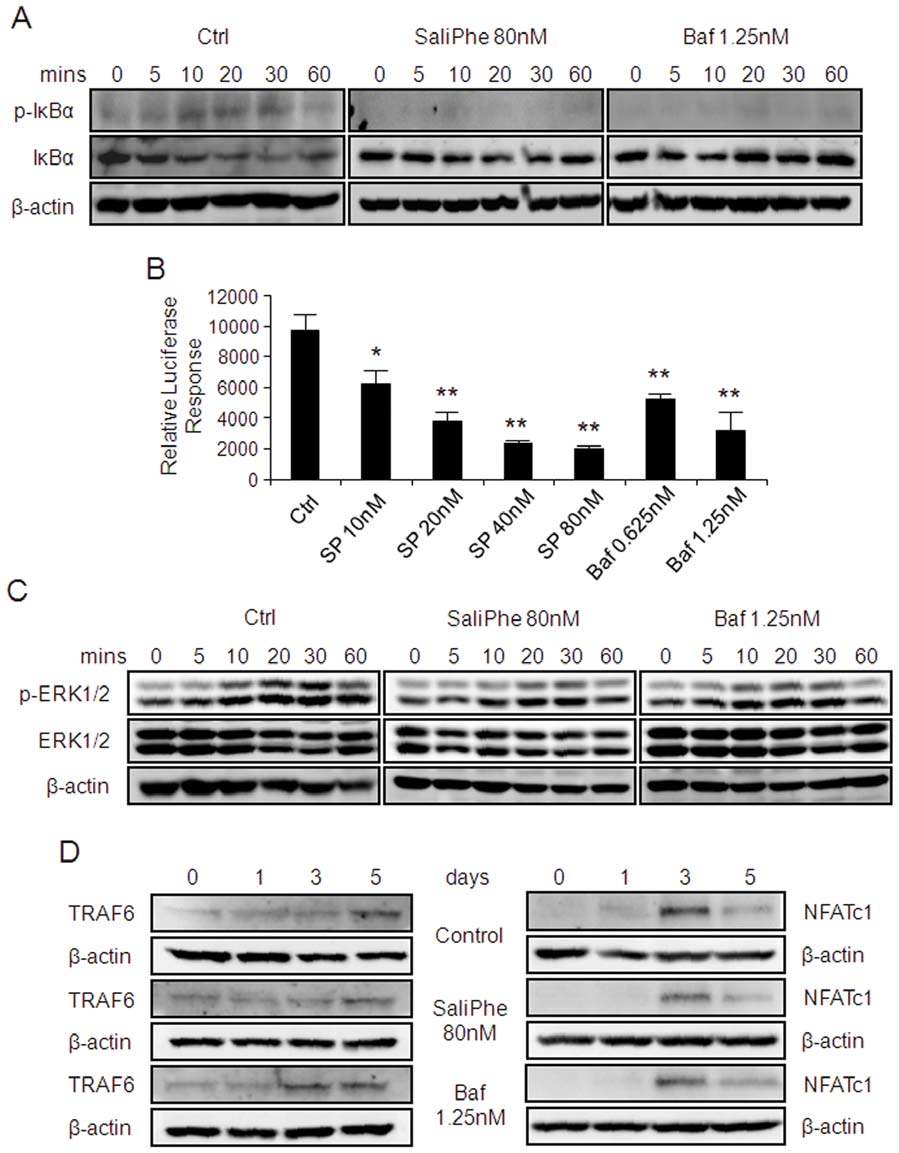
SaliPhe and bafilomycin impair the NF-κB and ERK1/2 signalling pathway. (**A**) The effect of saliPhe and bafilomycin on RANKL-induced IκBα phosphorylation and degradation. M-CSF-dependent BMMs pretreated with or without saliPhe (80 nM) or bafilomycin (1.25 nM) were stimulated with 100 ng/ml RANKL for the indicated duration. Cells were lysed and analysed by western blot using anti-p-IκBα and anti-IκBα antibody. Antibody against β-actin was used as loading control. (**B**) The effect of saliPhe and bafilomycin on NF-κB activation. RAW264.7 cells stably transfected with the p-NF-κB-TA-Luc luciferase reporter gene were pretreated with saliPhe (80 nM) or bafilomycin (1.25 nM) prior to stimulation with RANKL (100 ng/ml) for 8 hrs. Cell lysates were harvested and luciferase activity was measured. Relative luciferase response was plotted. Data representative of 3 independent experiments performed in triplicates (mean ± SEM; *P<0.05, **P<0.01 against control). (**C**) The effect of saliPhe and bafilomycin on ERK1/2 signalling pathway. BMMs pretreated with or without saliPhe (80 nM) or bafilomycin (1.25 nM) were stimulated by RANKL for the indicated duration. The expression of ERK1/2 and phosphorylated ERK1/2 were probed using corresponding antibodies. (**D**) The effect of saliPhe and bafilomycin on TRAF6 and NFATc1 induction. M-CSF-dependent BMMs pretreated with or without saliPhe (80 nM) or bafilomycin A1 (1.25 nM) was stimulated with 100 ng/ml RANKL for the indicated duration. Cells were lysed and analysed by western blot using anti-TRAF6 and anti-NFATc1 antibody. Antibody against β-actin was used as loading control.

To further explore the underlying mechanisms for the inhibitory effect of saliPhe and bafilomycin on osteoclast formation, we next examined the effects of these V-ATPase inhibitors on classical signaling pathways involved in osteoclastogenesis such as NF-κB, ERK and NFATc1. As expected, western blot analysis revealed that following RANKL stimulation, IκBα an inhibitory subunit of NF-κB is phosphorylated and rapidly degraded (within minutes of stimulation) (Fig. 6A, control). By comparison, IκBα degradation and phosphorylation were notably perturbed in the presence of either saliPhe or bafilomycin (Fig. 6A) implying a disruption in NF-κB activation. Attenuation of NF-κB activation was further confirmed by a luciferase reporter assay using a stable RAW264.7 (pseudo-osteoclastic) cell line carrying NF-κB luciferase reporter construct. Activation of the NF-κB luciferase reporter gene was demonstrated following RANKL stimulation and this activation was dose-dependently suppressed by both saliPhe and bafilomycin treatments (Fig. 6B; *P<0.05, **P<0.01). Additionally, RANKL-induced ERK1/2 phosphorylation was also attenuated by saliPhe and bafilomycin treatment (Fig. 6C). On the other hand, saliPhe and bafilomycin had little effect on TRAF6 and NFATc1 expression (Fig. 6D), suggesting that the inhibitory effect on osteoclast formation by the V-ATPase inhibitors is primarily through the suppression of NF-κB and ERK1/2 signaling pathways.

## Discussion

Periprosthetic osteolysis and subsequent aseptic loosening remains one of the most common complications limiting the long-term durability of TJA and is the major instigator underlying complex joint revision arthroplasty operations [1], [4], [41]. Extensive efforts have been made to improve the implant designs, biomaterials, and sterilization and surgical techniques. However, these approaches are unlikely to eliminate particle generation from bearing surfaces that stimulate a cascade of adverse biological reactions causing osteolysis and loosening that leads to failure of the total joint replacement [3], [4], [5], [6]. With our increasing understanding of the cellular and molecular events involved in particle-induced osteolysis, it has become apparent that development of novel strategies through pharmacological interventions is a viable option for the extension of the life span of TJA. Here, we demonstrate for the first time that the V-ATPase inhibitor saliphenylhalamide can attenuate wear particle-induced osteolysis in a mouse calvarial model. *In vitro* biochemical and morphological assays revealed that the inhibition of osteolysis is partially attributed to a disruption in osteoclast acidification and polarization, both are prerequisites for osteoclast bone resorption. Interestingly, saliPhe also impaired osteoclast differentiation via the inhibition of the NF-κB and ERK1/2 signaling pathways.

*In vivo* wear particle-induced mouse calvarial osteolysis was used as the model to explore the potential protective effect(s) of V-ATPase inhibitors during pathological bone destruction. Three dimensional reconstruction of the calvarial bone architecture by μCT, demonstrated that titanium (Ti) particles indeed induced significant middle suture bone destruction as compared to the controls. Remarkably, this particle-induced osteolysis was significantly reduced when mice were treated with saliPhe. Furthermore, histological assessments confirmed the Ti particle-induced osteolysis adjacent to the pronounced inflammatory reactive tissue with marked increase in TRAP-positive multinucleated cells. Treatment with saliPhe or bafilomycin attenuated the inflammatory reaction, bone destruction, and bone erosion area associated with particle-induced osteolysis. Collectively, these data demonstrate, for the first time, that saliPhe can be effectively used for the treatment of wear particle-induced osteolysis.

The molecular mechanism of action of saliPhe and bafilomycin is likely mediated by the inhibition of V-ATPase-mediated acidification, the essential process necessary for bone resorption. This is in line with *in vitro* analysis demonstrating inhibition of acridine orange fluorescence quenching, a sign of elevated cellular pH and impaired acidification by saliPhe and bafilomycin, the classical mode of action of V-ATPase inhibitors. In addition to V-ATPase-mediated acidification for bone resorption, osteoclasts must first undergo membrane polarization to form an F-actin ring/sealing zone surrounding the resorption area from the extracellular space. V-ATPase has also been implicated in modulating cytoskeletal F-actin assembly via association of the B and C subunits of the V1 domain of the V-ATPase complex with F-actin filaments [25], [42], [43], [44], [45]. Additionally, deletion of the C1 subunit severely disrupted the formation of the F-actin ring and this effect was proposed to be independent of the V-ATPase activity though the underlying mechanism which remains to be established [25]. Consistent with this non-canonical role in modulating F-actin assembly, we observed disruption of intact F-actin ring formation upon treatment of mature osteoclasts with either saliPhe or bafilomycin. Interestingly, the appearance of the F-actin rings disruption was morphologically distinct from the control group, though the precise reason of such discrepancy is presently unclear. Both saliPhe and bafilomycin treated cells formed irregularly small and more numerous F-actin rings. However, the limitation of our study on disruption of F-actin ring by the V-ATPase inhibitors is the lack of sufficient mechanistic data to demonstrate the indirect involvement of C1 and B2 of the V1 domain.

An interesting yet unexpected observation of the current study is the effect of V-ATPase inhibitors on RANKL-induced signaling cascades including ERK, JNK, p38, and transcription factor NF-κB [40]. V-ATPase has been shown to directly interact with TRAF6 which is a crucial upstream regulator of NF-κB signaling [46]. Our study has shown that both saliPhe and bafilomycin inhibited the phosphorylation and degradation of IκBα protein necessary for NF-κB nuclear shuttling and transcriptional activity essential for osteoclast formation, as well as ERK signaling pathway which plays a role in osteoclast formation, function and survival. NF-κB activation has also been directly linked to the development of prosthetic osteolysis during inflammation. Wear debris particles have been shown to activate NF-κB in various macrophage cell systems and induce the production of TNFα and IL-6 that modulates the inflammatory response as well as osteoclast formation [12], [18], [47], [48]. Thus, the potent inhibitory effect of saliPhe and bafilomycin A1 on Ti-particle induced osteolysis *in vivo* could possibly be a biphasic effect on inhibition of bone destruction and suppression of inflammatory response. However, further studies on the inflammatory process is required.

### Conclusions

We have demonstrated the therapeutic potential of the novel marine metabolite V-ATPase inhibitor, saliphenylhalamide on Ti particle-induced osteolysis. The mechanisms are likely attributed to the classical effect on V-ATPase-mediated bone resorption by osteoclasts, as well as non-canonical impairment of osteoclast activation and formation. Future studies are required to examine if V-ATPase inhibitors may also have an unexplored role in attenuating particle-induced inflammation.

## Materials and Methods

### Reagents

Saliphenylhalamide (saliPhe) was a kind gift from Prof. Jef K. De Brabander at Department of Biochemistry, University of Texas, Southwestern Medical Center (Dallas, USA). Bafilomycin was purchased from Sigma. Commercial pure titanium (Ti) particles, with average diameter of 4.50 µm, were purchased from Johnson Matthey and prepared as previously described [49]. The particles were sterilized by baking at 180°C for hrs, followed by treatment with 70% ethanol for 48 hr to remove possible endotoxin. The particles' endotoxin level was measured (<0.1EU/ml) using a commercial detection kit (Chromogenic End-Point TAL with Diazo Coupling Kit, Xiamen Houshiji, Fujian, PRC). GST-RANKL_160–318_ (rRANKL) recombinant proteins were expressed and purified in our laboratory as previously described [50]. Acridine orange was purchased from Sigma. Antibodies used include mouse monoclonal anti-β-actin (JLA20) (DSHB University of Iowa), rabbit polyclonal anti-IκBα (Santa Cruz), mouse monoclonal anti-p-IκBα (Santa Cruz), rabbit polyclonal anti-ERK1/2 (Promega), mouse monoclonal anti-p-ERK1/2 (Santa Cruz), rabbit polyclonal anti-TRAF6 (Santa cruz) and mouse monoclonal anti-NFATc1 (BD Pharmingen). All antibodies were used at the concentrations recommended by the supplier. Alexa Fluor 647-Phalloidin was from Molecular Probes (Invitrogen). Luciferase substrate was from Promega.

### Titanium particle-induced osteolysis mouse model

Wear particle-induced osteolysis model were generated as previously reported [51]. In brief, titanium particles were washed continuously in 100% ethanol for 48-hrs to remove adherent endotoxins, followed by resuspension in sterile PBS solution at a concentration of 300 mg/ml. A total of 30 8-week old C57BL/J6 mice divided into 6 groups of 5 mice were used. Thirty milligrams of titanium particles were embedded under the periosteum around the middle suture of the mice to induce bone destruction (5 groups). A group of mice was used as sham control. All procedures are in accordance with the official guidelines for animal care of the Shanghai Jiao Tong University School of Medicine (Shanghai, China; Animal Ethics Approval #201040). Two days post-implantation of titanium particles, indicated V-ATPase inhibitors or PBS were injected into the periosteum every other day for 14days before sacrifice and proceeded for further analysis. No adverse affects or fatalities were noted.

### Osteoclastogenesis and bone resorption

Adherent MCSF-dependent BMMs isolated from C57BL/J6 mice were seeded in a 96-well plate at density of 6×10^3^ cells per well and stimulated with 100 ng/ml rRANKL and 10 ng/ml M-CSF in the presence or absence of saliPhe (10 nM, 20 nM, 40 nM, and 80 nM) or bafilomycin (0.625 nM and 1.25 nM) for 5 days for the formation of multinucleated osteoclasts. After 5 days culture, cells were fixed in 4% paraformaldehyde and stained for TRAP activity. TRAP positive cells which contained 3 or more nuclei were scored as mature osteoclasts, and cell spread area were measured. BMMs derived preosteoclasts (3days osteoclastogenesis) were seeded on bovine bone discs and stimulated with rRANKL and M-CSF in the presence or absence saliPhe or bafilomycin as described. After 48 hours culture bone discs were fixed in 4% paraformaldehyde and stained for TRAP activity. The number of TRAP positive multinucleated osteoclasts (>3 nuclei) were quantified prior to assessment of resorptive activity. Resorption pits were visualized by scanning electron microscopy. ImageJ (NIH) software was used to quantify bone resorption parameters.

### Intracellular acidification by acridine orange staining

Intracellular acidification was determined by acridine orange (AO) fluorescence quenching method. BMM-derived osteoclasts treated with saliPhe or bafilomycin for 24 hrs were serum-starved for 2 hrs followed by incubation with 5 µg/ml AO (Sigma) for 30 mins at 37°C. Cells were washed and processed for fluorescent microscopy analysis on a NIKON Eclipse TE2000-S fluorescence microscope and associated software or fluorescence measured on FLUOStar Optima spectrophotometer (BMG LabTech) at excitation 492 nm and emission 535 nm. In addition, mature osteoclasts were also incubated with 5 µg/ml AO (Sigma) for 30 mins at 37°C and baseline acidification was recorderd prior to the addition of V-ATPase inhibitors and the effect on acridine orange quenching was chased under fluorescent microscope for 5 and 10 mins to study the change of intracellular acidification after V-ATPase inhibitors treatment.

### F-actin ring immunofluorescence

For F-actin ring immunofluorescent staining, saliPhe or bafilomycin treated osteoclasts cultured on bovine bone discs were fixed with 4% paraformaldehyde for 15 min and permeabilized for 5 mins with 0.1% v/v Triton X-100. Cells were incubated with Alexa-Fluor 647 phalloidin diluted in 0.2% w/v BSA-PBS for 1 hr, washed extensively with 0.2% w/v BSA-PBS and PBS, and finally mounted with ProLong Gold anti-fade mounting medium (Invitrogen). Detection of fluorescence was carried out on the NIKON A1Si spectral detector confocal system equipped with 40× (oil) lenses. Fluorescence images were collected using the systems NIS-C Elements software and analysed using ImageJ.

### Western blot

Total cellular proteins were extracted from cultured BMM cells using RIPA lysis buffer (50 mM Tris pH 7.5, 150 mM NaCl, 1% Nonidet P-40, 0.1% SDS, 1% sodium deoxycholate) supplemented with Protease Inhibitor Cocktail (Roche). Lysates were cleared by centrifugation at 16,000 g for 10 mins at 4°C and supernatant containing proteins were collected. For immunoblotting, extracted proteins diluted in SDS-sampling buffer were resolved by SDS-PAGE (10–15%) gels and then electroblotted onto nitrocellulose membranes (Hybond ECL, Amersham Life Science). Following transfer, membranes were blocked with 5% skim milk in TBS-Tween (TBS; 0.05 M Tris, 0.15 M NaCl, pH 7.5 and 0.2% Tween-20) for 1 hr and then probed with primary antibodies diluted in 1% (w/v) skim milk powder in TBS-Tween for 2 hrs. Membranes were washed and then incubated with HRP-conjugated secondary antibodies and antibody reactivity was detected by the Western Lightning Ultra Detection Kit (Perkin Elmer) using the FujiFilm LAS-3000 Gel Documentation System (FujiFilm) and its associated software.

### NF-κB luciferase reporter gene assay

To examine NF-κB activation, RAW264.7 cells stably transfected with a luciferase reporter gene p-NF-κB-TA-Luc [52], were plated in 24-well plate at a density of 1×10^5^ cells per well and pre-treated with saliPhe or bafilomycin A1 for 1 hr prior to rRANKL stimulation (100 ng/ml) for a further 8 hrs. Cell lysates were harvested and luciferase activity measured using the Promega Luciferase Assay System according the manufacturer's instructions (Promega). RAW264.7 subclone C4 was kindly provided by Dr. A.I. Cassady from the Centre for Molecular and Cellular Biology, Department of Biochemistry, University of Queensland.

### Micro-CT scanning

A high-resolution micro-CT (Skyscan 1072; Skyscan, Aartselaar, Belgium) was used to perform qualitative and quantitative analyses of osteolysis in mouse calvaria. In order to reduce metal artifact, the wear particles were removed before μCT scanning. A thickness of 9.1 um for each slice was used for scanning the whole calvaria. After reconstruction, a square region of interest (ROI) around the midline suture was chosen for further qualitative and quantitative analysis, with bone volume against tissue volume (BV/TV) and percentage of total porosity of each sample measured according to previous literatures [53]

### Histological and histomorphometric analysis

After μCT analysis, the samples were decalcified in 10% EDTA (pH 7.4) at 4°C for 2 weeks, followed by paraffin embedding. For the middle suture osteolysis area analysis, non-osseous tissue area adjacent and in continuity with the midline suture was taken as the osteolysis area, and calculated with Image Pro-Plus 5.0 (Media Cybernetics, USA). Five consecutive H&E stained sections were calculated. In addition, TRAP staining was performed for counting TRAP positive multinucleated osteoclasts in each sample.

### Semi-quantitative Reverse Transcription (RT)-PCR and Real-time qPCR

Total cellular RNA was isolated from cultured cells using RNeasy Mini Kit (QIAGEN) in accordance with the manufacturer's protocol. For RT-PCR, single-stranded cDNA was reverse transcribed from 2 µg total RNA using reverse transcriptase with oligo-dT primer. All PCR was carried out using 1 µl of each cDNA using the following cycling parameters 94°C, 40 secs; 60°C, 40 secs; and 72°C, 40 secs for 30 cycles with primers as: Atp6v1b2: Forward: ACGCTGAAGCGCTGCGAGAG; Reverse: CCTTCCACTCGACCGGCACG; Atp6v1c1: Forward: ACGCTCGCAGAAATGGTAGT; Reverse: TGCTTTCATCTCCTCCTCGT; 18S: Forward; ACCATAAACGATGCCGACT; Reverse: TGTCAATCCTGTCCGTGTC. PCR samples were analyzed by DNA agarose gel electrophoresis. For real-time qPCR analysis, corresponding PCR primers were used and performed on a 96-well plate ABI Prism 7000 Sequence Detection system (Applied Biosystems, USA) using SYBR Green PCR Master Mix (QIAGEN). Cycling conditions was as follows 95°C, 15 secs; 60°C, 20 secs; and and 72°C, 1 min for 40 cycles. The comparative 2^−ΔΔCT^ method was used to calculate the relative expression of each target gene and the expression level of all genes were normalized to the expression level of the house keeping gene.

### Bioluminescence Resonance Energy Transfer (BRET) Assay

BRET expression constructs were constructed as previously described [54]. Briefly, mouse sequence for V-ATPase V0 domain subunits *a3, c, c″*, *e* and accessory subunit Ac45 were fused to the mammalian expression vector pcDNA3.1 containing the *Renilla* luciferase (Rluc) donor fluorophore or EYFP-fused acceptor fluorophore. HEK293 cells were transiently co-transfected with both donor and acceptor constructs using Lipofectamine2000 transfection reagent in 6-well plates. Forty-eight hours post-transfection cells were harvest by trypsinization and ∼3×10^4^ cells per well were assayed in a 96-well plates in the presence or absence of V-ATPase inhibitors. Coelenterazine-h substrate (Promega) was added to a final concentration of 5 µM and readings immediately collected within the 475 nm (Rluc) and 530 nm (EYFP) windows after addition of the substrate using the PolarStar Optima Spectrofluorometer (BMG Labtechnologies) and its associated software. BRET ratios for the co-expression of Rluc and EYFP constructs were normalized against the control group. Data presented is representative of three independent experiments.

### Statistics & Data Presentation

Results were statistically analyzed using a two-tailed Students T-test using Microsoft Excel (Microsoft Corp.) and data shown represent one of at least three independent experiments.

## Supporting Information

Figure S1 **SaliPhe and bafilomycin do not alter V0 subunits interaction and V1 subunits B2 and C1 expression.** (**A**) The interaction of V0 domain subunits in the absence or presence of V-ATPase inhibitors by BRET assay. (**B**) Gene expression of V-ATPase subunit B2 and C1 in osteoclasts after saliPhe or bafilomycin treatment by RT-PCR. (**C**) qPCR for quantification of the expression of V-ATPase subunit B2 and C1 after V-ATPase inhibitors treatment.
(TIF)
