# Supplementary figures and images for: Prevention of Wear Particle-Induced Osteolysis by a Novel V-ATPase Inhibitor Saliphenylhalamide through Inhibition of Osteoclast Bone Resorption
Source: PLoS One. 2012 Apr 11;7(4):e34132. doi: 10.1371/journal.pone.0034132 (PMC3324493; doi:10.1371/journal.pone.0034132)

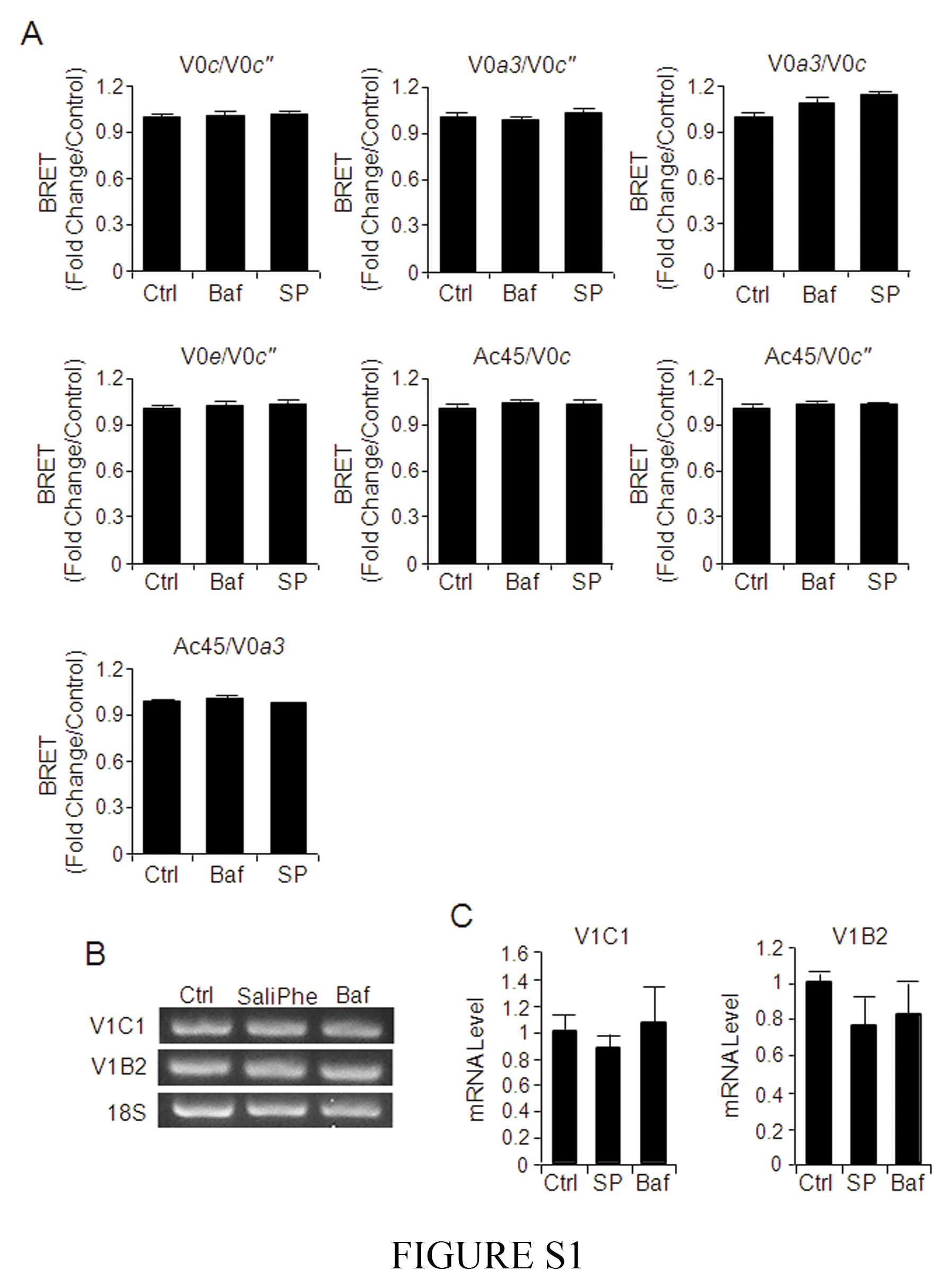

Supplement: Figure S1 — SaliPhe and bafilomycin do not alter V0 subunits interaction and V1 subunits B2 and C1 expression. (A) The interaction of V0 domain subunits in the absence or presence of V-ATPase inhibitors by BRET assay. (B) Gene expression of V-ATPase subunit B2 and C1 in osteoclasts after saliPhe or bafilomycin treatment by RT-PCR. (C) qPCR for quantification of the expression of V-ATPase subunit B2 and C1 after V-ATPase inhibitors treatment. (TIF) [file pone.0034132.s001.tif]
